# Exploring the Nonlinear Relationship between Renewable Energy Consumption and Economic Growth in the Context of Global Climate Change

**DOI:** 10.3390/ijerph192315647

**Published:** 2022-11-24

**Authors:** Yuting Feng, Tong Zhao

**Affiliations:** Business School, Nanjing Xiaozhuang University, No. 3601 Hongjing Avenue, Jiangning District, Nanjing 211171, China

**Keywords:** renewable energy consumption, economic growth, energy transition, sustainable development, global climate change

## Abstract

In recent years, the impact of global climate change has increasingly revealed that energy transformation has become an indispensable part of achieving carbon neutrality. Thus, the relationship between energy transformation and economic growth has become the focus of academic attention. This study examines energy transition issues by using the panel threshold method. It explores the nonlinear impact of renewable energy consumption on economic growth, identifies various factors that lead to this nonlinear impact, and verifies its threshold effect. A comprehensive analysis reveals the following. (1) Overall, renewable energy consumption inhibits real gross domestic product (GDP) growth, but, in the long run, the negative impact becomes positive. (2) The threshold effect of energy consumption intensity (EI) is significant, with a threshold value of approximately 3.213. This means that when EI ≤ 3.213, renewable energy consumption promotes economic growth. However, EI > 3.213 indicates that this impact is significantly negative, which means that advancing the energy transition at this time may occur at the expense of real GDP growth. (3) There is also a significant threshold effect in energy transformation, with a threshold value of approximately 6.456. Similarly, when energy consumption transition (ET) ≤ 6.456, renewable energy consumption dampens real economic growth, and the economic cost of promoting renewable energy consumption is greater at this time. Alternatively, when ET > 6.456, this impact is significant at the 1 percent level and significantly positive. (4) There is also a significant threshold effect for emerging technologies, with a threshold value of approximately 1.367. When ET ≤ 1.367, renewable energy consumption dampens real economic growth, and the economic cost of promoting renewable energy consumption is greater. When ET > 1.367, the impact is significantly positive at the 1% level. To promote the positive development of economic growth, climate change, and energy transition, the nonlinear relationship studied in this paper can fill the gaps in existing research in theory and provide a theoretical basis for the government to adopt different policies at different stages of the energy transition to lay the foundation for improving global climate change in practice.

## 1. Introduction

The key role of energy has received considerable attention. Environmental problems have become increasingly prominent in the context of economic growth. The contemporary energy problem is not only about how to provide a safe energy supply for economic growth but also about how to control the greenhouse gas emissions associated with the massive combustion of traditional energy. Advances in technology have enhanced the potential for utilizing renewable energy. As an alternative to traditional energy, renewable energy has become a key development area for countries worldwide because of its clean, low-carbon, and sustainable advantages [1,2]. According to the 2019 edition of the World and China Energy Outlook 2050, clean energy will gradually replace coal, and the proportion of clean energy is expected to reach 56% by 2050 [3,4]. With rapid economic growth, the demand for energy in various countries is also increasing, resulting in traditional fossil energy depletion and more serious problems, such as environmental pollution [5,6,7]. To ensure national energy security, countries worldwide have turned their attention to renewable energy and listed it as part of their national security policies. The global energy structure has undergone several major changes. Although fossil energy still occupies a large proportion, such a proportion is showing a downward trend, while the renewable energy proportion is increasing. This pattern indicates that the energy consumption structure is gradually transforming into renewable energy consumption. Since the beginning of the 21st century, energy conservation, emission reduction, and green and low-carbon energy have been greatly emphasized globally, while the global renewable energy industry has undergone rigorous development and received increasing attention. Countries regard increasing renewable energy consumption and promoting energy transition as an important energy strategy [8,9,10].

China’s current energy consumption still follows the multi-energy complementary model, in which “fossil energy is the main source of energy, and the proportion of renewable energy is relatively small.” The country’s promotion of energy transition involves external and internal driving forces. Externally, as an active participant in global climate governance, China publicly announced its willingness to accept post-2020 global emission reduction targets as early as the 2011 Durban Climate Conference and formally proposed national autonomy at the 2015 Paris Climate Conference. In terms of its contribution target (Intended Nationally Determined Contributions), the country considers greenhouse gas emissions to peak by 2030 and decrease by 60% to 65% by 2050 and expects renewable energy consumption to reach approximately 20%. It has thus emphasized that it will optimize the energy structure through internal adjustment, comprehensively promote the energy transformation strategy, and ensure the realization of the nationally determined contribution target. The problem of smog has become a topic of great concern among the whole population, and there has been a consensus within the country to change the traditional energy consumption mode dominated by coal. In 2017, “Resolutely Fighting the Blue Sky Defense War” was directly included in the government’s report, emphasizing that the problem of air pollution prevention and control should be completely solved by controlling the total consumption of coal and increasing the clean energy proportion. At present, there is an urgent need for environmental protection and the stimulation of energy transition. In this context, the vigorous development of the renewable energy industry is the main way to achieve carbon neutrality. Achieving renewable energy to meet future energy demands has become the focus of energy transition [11,12]. In other words, renewable energy has become the core logic element of China’s energy transition strategy, and it serves as the basic starting point for the current study to discuss energy transition. Renewable energy refers to energy that produces little or no pollutants during development and utilization [13,14]. At the Paris Climate Conference in December 2015, China put forward a specific goal of “peak CO_2_ emissions and 20% renewable energy consumption by 2030.” Given such demanding goals, the following three questions should be addressed:

- Will energy transition compromise economic growth?
- Can renewable energy consumption promote energy transition and consequently impact China’s economic growth?
- What are its determinants? This topic deserves further discussion to provide a theoretical basis for China’s energy transition.

## 2. Literature Review

With the development of the renewable energy industry and the increasing attention paid to energy transition by countries around the world, academia has recognized the role of renewable energy. Renewable energy is subdivided into renewable energy and nonrenewable energy consumption, which are applied to production functions to analyze their respective effects on economic growth [15,16,17]. Currently, the literature in this area offers the following three conclusions.

### 2.1. The Increase in Renewable Energy Consumption Promotes Economic Growth

Some scholars believe that renewable energy consumption, together with other factors, promotes economic growth. Relevant research indicates that renewable energy consumption can improve economic levels [18]. Khan et al. [19] pointed out that while renewable energy consumption promotes the economy, the economy positively drives renewable energy consumption by promoting green technology. Topcu and Tugcu [20] found that the industry could generate new jobs and solve the unemployment problem. In turn, economic growth can be promoted, and green and high-quality economic growth can be achieved. On this point, foreign scholars Apergis and Salim [21] and Markandya et al. [22] believe that employment can promote economic growth. Zafar et al. [23] and Kocak and Sarkgunesi [17] found that reducing fossil fuel energy consumption has a significant effect on economic growth. Odhiambo [24] and Naseri et al. [25] concluded that energy consumption can drive gross domestic product (GDP) growth. Zrelli [26] selected Mediterranean countries from 1980 to 2011 for analysis and concluded that a two-way Granger causality exists between electricity consumption and economic growth and that renewable energy consumption is the most important reason behind economic growth.

### 2.2. The Increase in Renewable Energy Consumption Compromises Economic Growth

Some findings suggest that renewable energy consumption hinders economic growth [27]. This is because of the economic costs incurred in transforming production methods in the process of using renewable energy to replace fossil energy. Maji et al. [28] believe that renewable energy consumption reduces the total factor productivity of society, thereby reducing the speed of economic growth. Han et al. [29] also believe that renewable energy consumption is not conducive to business production efficiency as it reduces firms’ profitability. Bhattacharya et al. [30], on the basis of annual data from to 1991 to 2012, found that in India, Ukraine, the United States, and Israel, renewable energy consumption inhibits economic development. Scholars Ocal and Aslan [31] arrived at the same conclusion, explaining that the relationship between the two changes is contradictory. Qi and Li [32] found that China lacks technological advantages in renewable energy development and that the related cost of use is relatively high. If the proportion of renewable energy increases, then economic growth may decrease.

### 2.3. Renewable Energy Consumption Has No Significant Impact on Economic Growth

The existing research results also show that the relationship between renewable energy consumption and economic growth is not significant. For example, Payne [33] found no Granger causality between the two elements in the United States, and Menegaki [34] reported the same in the context of Europe, possibly because the proportion of this industry is relatively low and the impact on economic growth is small and insignificant. Bao and Xu [35] considered China’s provinces as research objects, analyzed the spatial heterogeneity of the causal relationship between them, and concluded that more than half of the regions lack a causal relationship. Chang et al. [36] and Bulut and Muratoglu [37] also supported the conclusion of a neutral relationship between renewable energy consumption and economic growth on the basis of different country or regional samples, time spans, and econometric models. Yu and Hwang [38] extended the time series data interval of Kraft and Kraft [39] by three years and verified the conclusion of Akarca and Long [40] that the relationship is different from that reported by Kraft and Kraft. In sum, no causal relationship was found. Yu and Jin [41] continued to expand the time series of the study on the basis of their predecessors and used the Engle–Granger two-step method to analyze and overturn the research results of Kraft and Kraft, arguing that income and energy consumption are related; the authors found no causal relationship between the two variables.

### 2.4. Regional Differences in the Impact of Renewable Energy Consumption on Economic Growth

It has been found that there are regional differences in the impact of renewable energy consumption on economic growth (or employment). Al-Mulali et al. [42] find that the positive impact of renewable energy consumption on economic growth is more persistent and significant at higher income levels. Yao and Zhang [43] and Yan et al. [44] find that there is heterogeneity in the impact of renewable energy consumption on employment in different regions. Qi and Li [32] found that the impact of renewable energy consumption growth on economic growth in EU member states with different levels of economic development was in the opposite direction. Guo and Cai [45] explored the nonlinear correlation mechanism between transport, tourism development, renewable energy, and economic growth. The results show that there is significant regional heterogeneity in the moderating effect of renewable energy. Chen and Ye [46] showed that there are large regional differences in economic growth and electricity production from traditional and renewable energy sources in China.

In summary, the main views of statistical scholars in this paper are listed in Table 1 below.

Combined with the above, it can be seen that there are more research results on the relationship between the two, which provides a great deal of reference material for this paper and helps the innovation of this paper. Compared with the previous scholars’ research, the innovation of this paper is reflected in the following two aspects. 

(1) From the theoretical research, the existing research lacks an exploration of the nonlinear influence relationship of energy transition; meanwhile, there are fewer studies that carry out nonlinear research on renewable energy transition and economic growth in the context of global climate—the authors can only retrieve one paper by searching the keywords of “renewable energy” and “nonlinear” on the China Knowledge Network; with the keyword of “renewable”, the authors can retrieve 31 papers by searching “renewable energy” and “economic growth”, and four papers by searching “energy transition” and “economic growth”. In the Web of Science database, 178 papers can be retrieved with the keywords of “renewable energy” and “economic growth”, and only one paper can be retrieved with the keyword of “nonlinear relationship”. Through the search of the existing literature, we can find that although there are more studies on the relationship between energy transition and economic growth, there are very few studies on the nonlinear relationship, so this study can fill the gap in existing theoretical studies.

(2) From the perspective of practical value, countries around the world cannot avoid the serious problems brought by climate change, the depletion of fossil energy, and ecological environment destruction. In the face of these problems, countries around the world are taking active approaches to address these issues in terms of energy production and energy consumption. Energy transition, with clean energy as the core energy source, is being promoted in most countries, and energy transition and development is a major issue related to the sustainable development of the economy, society, and environment. Whether from the perspective of total energy consumption control, energy consumption restructuring, downward carbon emission growth rates, etc., the acceleration of energy transition cannot be delayed. In this context, it is of great significance to study how to achieve sustainable economic growth through energy transition. The nonlinear relationship between energy transition and economic growth can provide a theoretical basis for the government to take decisions at different stages, and thus this study has certain guiding significance for policy practice.

## 3. Methods

### 3.1. Theoretical Models

When analyzing linear econometric models, empirical studies are generally conducted using cross-sectional data or time series data. Since the 1950s, with the development of econometric theory, panel data models have emerged to solve the above problems.

Panel data have the characteristics of both cross-sectional data and time series data, and they are two-dimensional data obtained in time and space; a model built with panel data is called a panel data model. Compared with classical econometric models, panel data can not only reflect the variation patterns and characteristics of variables in both time and cross-sectional dimensions, but also have the following advantages: first, they can build more comprehensive models to meet the needs of actual economic analysis; second, classical econometric regression models are prone to multiple cointegration problems, but panel models can effectively reduce the emergence of such problems; third and fourth, panel data can effectively control individual heterogeneity; fifth, they can identify some problems that cannot be detected by time series analysis and cross-sectional analysis, and this reduced the bias of omitting important explanatory variables.

The panel data model can make comprehensive use of the sample data in many aspects and reflect the trends and patterns of changes in the sample variables from both time and space dimensions, which has an irreplaceable role in quantitative economic analysis and has high application value and greatly enriches the practicality of the panel data model. The panel data model is a linear regression model, and the basic expression of the model takes the form as follows:

(1) $$y_{it}=\alpha_{it}+\sum_{k=1}^{p}\beta_{kit}x_{kit}+\mu_{it}\;\;\left(i=1,2,\cdots ,n;t=1,2,\cdots ,T\right)$$

where $y_{it}$ is the observed value of the dependent variable *y*; $\alpha_{it}$ is the intercept of cross-sectional individuals; $\beta_{kit}$ is the marginal value; *n* is the number of cross-sectional individuals; *T* is the sample size of each interface individual time series; *P* is the number of explanatory variables, and the model is dynamic if it contains lagged variables of the dependent variable—otherwise, it is a static model; $\mu_{it}$ is the random disturbance term.

### 3.2. Unit Root Test of Panel Data

Panel data models require stationary panel variables; otherwise, spurious regressions may occur. Therefore, a unit root test should be performed on panel variables before building a panel data model. Panel variable unit tests can be divided into two categories.

(1) Unit root test for homogeneous roots

The test methods include the Levin–Lin–Chu (LLC), Breitung, and Hadri tests for homogeneous roots. The LLC test is based on the augmented Dickey–Fuller (ADF) test. The null hypothesis is that the same unit root exists. The Breitung test is similar to the LLC test; however, the proxy variables in the test formula are different. The null hypothesis of the Hadri test is that no section sequence contains a unit root.

(2) Unit root test for heterogeneous roots

Heterogeneous root tests include the Im, Pesaran, and Shin (IPS) test; the Fisher-ADF test; and the Fisher–Phillips–Perron test. The construction of the heterogeneous root test statistic combines the results of each section of the data and finally provides a comprehensive conclusion on whether the individual section has a unit root. The test is as follows:

(2) $$y_{it}=\varphi_{i}y_{it-1}+x_{it}^{\prime }\beta_{it}+\mu_{it}\;i=1,2,\cdots N;t=1,2,\cdots ,T$$

where $x_{it}$ is the exogenous variable; $\beta_{it}$ represents the regression coefficient; *N* represents the sample number; *T* represents the time length; $\mu_{it}$ represents the random disturbance term; and $\varphi_{i}$ is the coefficient of $y_{it-1}$ in the autoregressive equation, where |$\varphi_{i}$| < 1 means that the original sequence is stationary and |$\varphi_{i}$| = 1 means that the original sequence is nonstationary.

### 3.3. Cointegration Test

After passing the unit root test, if the cointegration test conditions are met, the cointegration relationship between variables needs to be further verified to determine the short- and long-term cointegration relationship. Therefore, a panel cointegration test is required. Cointegration test methods include the residual-based Lagrange multiplier test derived by McCoskey and Kao; the DF and ADF unit root tests proposed by Kao [47]; the likelihood-based test proposed by Larsson, Lyhagen, and Lothgren; and the co-integration test of heterogeneous panel data proposed by Pedroni [48]. The Kao and Pedroni tests are the most widely used by domestic scholars. They are also used in the current work to examine the cointegration relationship between variables and determine whether further regression estimation can be performed.

### 3.4. Panel Threshold Model

Generally, panel data are subject to heterogeneity. Every individual in an actual study is different, and structural relationships among different variables may behave differently. Traditional fixed-effect or random-effect models reflect heterogeneity only in the intercept term. Threshold effect theory assumes that a linear relationship in the traditional concept does not exist and that variables reflect a nonlinear relationship because of differences in their development. When an independent variable is in the initial stage of development, the dependent and independent variables maintain a specific interaction relationship. When the independent variable reaches a certain threshold, the dependent and independent variables demonstrate another interaction relationship. Only when the amount of influence reaches a certain range of change will this relationship exist as a nonlinear one. Hansen [49] first proposed the panel threshold model to evaluate and detect the threshold value and effect and select the explanatory variable to be judged. In this model, a piecewise function is built to detect threshold values and effects through estimation and judging. One of the main advantages of this model is that it eliminates the need to set the nonlinear equation shape and that the threshold value is objectively confirmed by the threshold value of the sample; thus, the error caused by the subjective area division can be eliminated. In the current study, the threshold panel regression method developed by Hansen [50,51] can be used to obtain the endogenous threshold value and estimate the statistical significance of the threshold value. The threshold effect means that when renewable energy consumption reaches the critical threshold value, economic growth will show a relationship that differs from that when the consumption does not reach the threshold.

#### 3.4.1. Panel Model Construction

In this study, the production function that has been widely used in western economics—that is, the Cobb–Douglas function—is selected as the theoretical model [52]. Since the introduction of this production function, the academic community has continuously produced relevant research results, thereby enriching the research and application of such a function. The function is as follows (3):

(3) $$Y=f\left(L,K\right)=AL^{\alpha }K^{\beta }$$

where *Y* represents the economic output; *K* represents the capital stock; *L* represents the labor input; *A* represents the production technology factor; and *α* and *β* are the output elasticity coefficients of the labor input and capital stock, respectively. Output elasticity refers to the relative change in the ratio of output to input; it represents each input factor’s contribution to economic growth, thus reflecting the relationship between them.

This study improves the traditional Cobb–Douglas production function, subdivides energy into renewable energy and nonrenewable energy [53,54,55], and then adds them to the Cobb–Douglas production function together with the production factors that affect economic growth, such as capital and labor. The indicator selection of Li and Xu [56] is considered in the selection of renewable energy consumption (REC), nonrenewable energy consumption (NREC), labor input (*L*), and capital (*K*) as independent variables and real GDP(*Y*) as a dependent variable. The extended Cobb–Douglas production function can be expressed as

(4) $$\;Y_{it}=f\left(K_{it};L_{it};REC_{it};NREC_{it}\right)\;$$

Here, *i* represents the area, while *t* represents the time; to eliminate the influence of the time factor on the equation, the logarithm of both sides of the equation is taken, and the error term $\varphi_{it}$ is added to obtain the following equivalent regression equation:

(5) $$\ln Y_{it}=\beta_{1i}lnK_{it}+\beta_{2i}lnL_{it}+\beta_{3i}lnREC_{it}+\beta_{4i}lnNREC_{it}+\varphi_{it}\;$$

$\beta_{1i}$, $\beta_{2i}$, $\beta_{3i}$, $\beta_{4i}$ respectively indicate the factor output elasticities. The equation is a pair of Cobb–Douglas production functions that include REC, NREC, *L*, and *K* as factors in the production number model.

Next, production Equation (5) is further extended. The basic form of the constructed regression model is (with a single threshold as an example)

(6) $$\ln Y_{it}=\beta_{0}+\beta_{1i}lnY_{i,t-1}+\beta_{2i}lnX_{it}\cdot I\left(q_{it}\leq \gamma \right)+\beta_{3i}lnX_{it}\cdot I\left(q_{it}>\gamma \right)\;+\sum \beta_{ni}Z_{it}+\mu_{i}+v_{t}+\varepsilon_{it}\;$$

where $lnX_{it}$ represents the core explanatory variable. In this work, renewable energy is denoted as $lnREC_{it}$. Other explanatory variables include $lnY_{i,t-1}$ and $Z_{it}$, with $Z_{it}$ being a matrix of control variables. *β_ni_* is a coefficient, while $q_{it}$ represents the threshold variable corresponding to the three variables of energy transition, energy intensity, and new technology level selected in this study. γ is the corresponding threshold value, which divides the entire sample into two groups; the corresponding coefficients are $\beta_{2i}$ and $\beta_{3i}$. *I* (•) is an indicator function. $\mu_{i}$ and $v_{t}$ are the unobservable individual fixed and time effects, respectively. $\varepsilon_{it}$ is a random disturbance term that obeys an independent and identical distribution.

#### 3.4.2. Estimation of Dynamic Panel Threshold Model

For any given threshold *γ*, *β* can be estimated by least squares estimation.

(7) $$\hat{\beta }\left(\gamma \right)={\left(X^{*}{\left(\gamma \right)}^{\prime }X^{*}\left(\gamma \right)\right)}^{-1}X^{*}{\left(\gamma \right)}^{\prime }Y^{*}\;$$

The residual is

(8) $$\;{\hat{e}}^{*}\left(\gamma \right)=Y^{*}-X^{*}\left(\gamma \right)\hat{\beta }\left(\gamma \right)\;$$

The residual sum of squares is

(9) $$S_{1}\left(\gamma \right)={\hat{e}}^{*}{\left(\gamma \right)}^{\prime }{\hat{e}}^{*}\left(\gamma \right)=Y^{{*}^{\prime }}{\left(I-X^{*}{\left(\gamma \right)}^{\prime }X^{*}\left(\gamma \right)\right)}^{\prime }X^{*}{\left(\gamma \right)}^{\prime }Y^{*}\;$$

(10) $$\hat{\gamma }=argmin_{\gamma }S_{1}\left(\gamma \right)\;$$

Once the threshold value γ is determined,

(11) $$\hat{\beta }=\hat{\beta }\left(\hat{\gamma }\right),{\hat{\sigma }}^{2}=\frac{1}{n\left(T-1\right)}{\hat{e}}^{*\prime }{\hat{e}}^{*}=\frac{1}{n\left(T-1\right)}S_{1}\left(\hat{\gamma }\right)\;$$

#### 3.4.3. Significance Test of Threshold Effect (Nonlinear Test)

Hansen [49] pointed out that the estimation has two basic assumptions. In the first assumption, the original hypothesis is H_0_: *β*_2_ = *β*_3_, meaning that there exists a linear relationship; the alternative hypothesis is H1: *β*_2_ ≠ *β*_3_, which signifies the existence of a threshold effect. The test statistic is $F_{1}=\left(S_{0}-S_{1}\hat{\gamma }\right)/{\hat{\sigma }}^{2}$, where $S_{0},\;\;S_{1}\hat{\gamma }$ are the residual sums of squares under assumptions H_0_ and H1, respectively. Hansen suggested using bootstrapping to obtain the distribution of F to obtain an effective *p* value. When *p* ≤ α, H_0_ is rejected, indicating the existence of a threshold value; α represents the significance level.

As for the second assumption, the threshold estimator is equal to the true value. When H_0_ is established, *β*_2_ = *β*_3_, indicating no threshold effect; the model degenerates into a linear model. If H_0_: *β*_2_ = *β*_3_ is rejected, it can be considered to have a threshold effect; then, the threshold estimate authenticity can be tested, H_0_: $\hat{\gamma }$ = γ0, H_1_: $\hat{\gamma }\;$≠ γ0. Hansen uses the maximum likelihood test threshold value, and the test statistic is $LR_{1}\left(\gamma \right)=\left(S_{1}\left(\gamma \right)-S_{1}\hat{\gamma }\right)/{\hat{\sigma }}^{2}$. $S_{1}\left(\gamma \right),\;\;S_{1}\hat{\gamma }$ are the residual sums of squares under the assumptions H_0_ and H_1_, respectively. The distribution of this statistic is nonstandard. When *LR*_1_ (γ0) ≤ c (α), H_0_ is rejected, and the threshold estimator is the true value. Among them, $c\left(\alpha \right)=-2\left(1-\sqrt{1-\alpha }\right)$, and α is the significance level.

### 3.5. Variable Selection and Description

(1) Explained variable: Economic growth (*Y*)

This study directly selects real GDP (*Y*, unit: 100 million yuan) to measure economic growth. It is obtained by deflating the price GDP and GDP index of the year published in the statistical yearbooks of various provinces over the years, taking 2008 as the base period of real GDP.

(2) Explanatory variables

Renewable energy consumption. Renewable energy refers to energy sources that can be recycled in nature on their own. The consumption of renewable energy is represented by water, solar, and wind energy, and the annual consumption of these three types of energy is summed up for each region as the renewable energy consumption data. According to the BP World Energy Statistics Report, the renewable energy consumption data are based on electricity generation, so the unit of renewable energy consumption can be trillion watt hours (TWh).

Nonrenewable energy consumption. Non-renewable energy consumption, which mainly includes data on oil, natural gas, coal, and nuclear energy consumption, is summed up for energy data as nonrenewable energy consumption data. According to the description of the BP World Energy Statistics Report, the consumption data of coal only consider commercial solid fuels, including anthracite and bituminous coal and other stationary coal fuels, etc.; the consumption data of natural gas do not consider the part converted into liquid fuels, but include gas derivatives of coal (such as gas, etc.); the consumption data of nuclear energy are based on the conversion of electricity generation.

Capital stock (*K*). We use annual capital formation and annual capital stock to express capital stock. We adopt the capital stock calculation formula for the estimation:

(12) $$\;K_{t}=I_{t}+\left(1-\delta_{t}\right)*K_{t}$$

(13) $$K_{0}=I_{0}\left(1+g\right)/\left(g+\delta \right)$$

where *t* is the year, *K* is the capital stock, *I* is the investment, and *δ* represents the depreciation rate. $K_{0}$ represents the initial capital stock, $I_{0}$ represents the capital investment amount, and *g* is the average growth rate of fixed asset investment. This study sets the depreciation rate at 4% [57].

Labor force (*L*). The data in this study are annual national employment data from 2008 to 2020.

(3) Threshold variable

Energy consumption intensity (EI). EI is measured by dividing the energy consumption of each province by the total GDP. Energy consumption intensity is defined as the ratio of the total converted value of energy consumption to a country’s GDP over a certain period of time. It measures the dependence on energy sources. When this ratio reaches a certain height, the degree of energy consumption is correlated with the economic growth rate. Therefore, with faster economic growth, promoting an energy transition by increasing renewable energy consumption requires a higher economic price [58]. Therefore, we propose the following.

**Hypothesis** **1.** *The impact of renewable energy consumption on economic growth has a threshold effect on energy consumption intensity. When renewable energy consumption is in the high threshold range, it will have a negative impact; when it is in the low threshold range, it will have a positive effect*.

Energy consumption transition (ET). This refers to the process in which fossil energy consumption is still dominant, but renewable energy consumption is developing rapidly and is gradually replacing fossil energy consumption [59]. This study uses the proportion of renewable energy consumption to reflect the degree of energy consumption transformation. This study divides energy transition into three stages: initial, maturity, and overmaturity. First, in the early stage of transformation, the degree of energy transformation is low, and the proportion of renewable energy consumption is small; however, it has not yet formed a scale, and its effect on reducing carbon emissions is limited. At the same time, research and development (R&D) investment in related technologies in the early stage of transformation is large, the cost is high and immature, and the use efficiency is low, thereby imposing a possible burden on economic growth. Over time, after the renewable energy industry gradually matures in terms of technology and management, the energy transformation enters a mature period and reaches a certain level, and its effect on reducing carbon emissions increases significantly. Therefore, we believe that there is a threshold energy transformation effect, which we should pay attention to. Hence, the following hypothesis is proposed.

**Hypothesis** **2.** *The transformation of the energy consumption structure can only be beneficial to the promotion of economic growth to an appropriate extent*.

Emerging technologies (NT). Emerging technologies can lead to an increase in a society’s productivity. Emerging technologies can improve production efficiency by optimizing production processes [60,61,62,63]. This study expresses the number of new technology patents in the renewable energy industry as a percentage of total energy industry patents. Renewable energy technology patents refer to the number of EPO patents applied worldwide. In this paper, we mainly select emerging technology patents in the field of renewable energy, and, from the data available, we mainly select patents obtained in the fields of solar energy, hydro energy, and wind energy. Emerging technologies usually have two paths for technological progress. The first is completely innovative technology, which is characterized by the emergence of technologies that promote a substantial or even revolutionary increase in social productivity. The second is the upgrade of existing technology, which is characterized by improving and optimizing the existing technology to achieve the effect of improving social productivity. As emerging technologies can promote renewable energy utilization efficiency without negatively affecting economic growth, it is necessary to focus on developing emerging technologies.

**Hypothesis** **3.** *Accelerating technological innovation and obtaining and using cutting-edge technologies can effectively enhance the level of economic growth during the energy transition and break through the existing threshold effect*.

### 3.6. Data Description

The original data selected in this study mainly come from the EPS database, “China Statistical Yearbook”, “China Economic and Social Development Statistical Database”, and the statistical yearbooks of various provinces in China. According to the availability of data, this study selects the provincial panel data of 30 regions from 2008 to 2020 (autonomous regions and municipalities, excluding Tibet). The descriptive statistics of the variables are listed in Table 2.

## 4. Results and Discussion

### 4.1. Unit Root Test

In general, if the sequence becomes stationary after *d* differences, the time series is called a d-order single integral sequence. If a series cannot be made stationary regardless of the number of differences, then the series is a non-single integral series. Unit root tests include the LLC, IPS, Fisher-ADF, and Fisher-PP tests, and they are performed on the variables. In this study, the panel unit root LLC, IPS, Fisher-ADF, and Fisher-PP tests are used to test the stability of each variable; the results are shown in Table 3. The variables in Table 2 are first-order difference variables, and they all reject the null hypothesis. Therefore, each variable is a first-order stationary sequence, I (1), which means that further cointegration tests can be performed.

### 4.2. Panel Cointegration Test

As the unit root test is stationary, we use the cointegration test method proposed by Pedroni [48], who constructed four “joint within” statistics and three “between” statistics. These seven statistics asymptotically obey the normal distribution of (0, 1) and provide critical values. If the calculated statistic is above the critical value, H_0_ is rejected, implying that there exists a long-term cointegration relationship. The null hypothesis of the Kao test is that there is no cointegration relationship. We mainly observe the t-statistic and the corresponding *p* value in the results. The judgment method for the size of the *p* value is consistent with the judgment method in the unit root test. Pedroni and Kao’s panel cointegration tests are performed on the variables, and the lag order is determined using the Schwarz information criterion. Table 4 presents the results. In the Kao test, the ADF statistic passes the significance test. In the Pedroni test, all statistics pass the significance test. This result shows that the explanatory variable of environmental pollution and other explanatory variables have a long-term cointegration relationship.

### 4.3. Estimation and Authenticity Test of Threshold Value

According to the threshold method, an empirical analysis is conducted on the impact of renewable energy on economic growth under energy transition. First, we test the threshold effect and set the existence of a single threshold and double threshold to carry out the self-sampling test and observe the number of thresholds. Finally, the estimated threshold value is obtained. F and *p* values are obtained after 500 repeated samplings (Table 5). The results show that only the single threshold model passes the significance test, while the double threshold effect model does not.

In Table 5, we find that no threshold effect is rejected; thus, there exists a nonlinear relationship in the model, and EI, ET, and NT are reasonable for establishing dynamic panel threshold models with threshold variables.

### 4.4. Estimation Results of Dynamic Panel Threshold Model

On the basis of the threshold effect determination, this study selects three different threshold variables for the regression analysis of the threshold model. The system Gaussian mixture model (GMM) is used for the regression estimation. The results are shown in Table 6.

(1) From the perspective of linear models

The impact of renewable energy consumption on real GDP is negative—that is, the energy structure transformation that increases renewable energy consumption can inhibit economic growth. From the perspective of the system GMM and threshold models, the regression estimation results are relatively robust. The main reasons are as follows. First, increasing renewable energy consumption has no technical or cost advantages at present. Renewable energy has developed rapidly in developed countries. In developing countries, government investment is increasing, and its development is gradually promoted through the guidance of the government. However, the huge financial expenditure of the government also has a negative impact on the economy, resulting in a certain economic cost. At present, many regions in China still rely on traditional fossil energy. The government has obtained more economic benefits from fossil energy, which has promoted economic growth, thus crowding out the development of renewable energy. Meanwhile, investment in technological innovation is insufficient. However, in the long run, with the improvement of technological innovation capability and the gradual expansion of the market scale, this negative effect will gradually turn into a positive effect.

(2) Regression results of the threshold model

First, this impact exerts an energy consumption intensity (EI) threshold effect with a threshold value of approximately 3.213. When EI ≤ 3.213, there is a significant positive impact; when EI > 3.213, the impact is significantly negative. This is mainly because under a high energy transition intensity, economic growth is highly reliant on energy consumption. At this time, increasing renewable energy consumption will incur a greater cost—that is, the reverse change between the energy substitution effect and energy consumption intensity. Higher energy consumption intensity indicates a lower substitution effect. Currently, the replacement of elements between renewable and nonrenewable energy is relatively difficult, and increasing renewable energy means a higher economic cost. The two mechanisms will vigorously develop the renewable energy industry at the expense of economic growth when the energy consumption intensity is higher than a certain threshold. A lower energy consumption intensity also corresponds to a lower energy dependence effect or higher energy use efficiency. At this time, the vigorous development of the renewable energy industry will promote economic growth. Therefore, it is necessary to implement different countermeasures for the energy consumption intensity of different regions. For example, regions with low energy consumption intensity can increase their renewable energy consumption, promote renewable energy industry development, and accelerate energy transformation, all of which exerts a positive impact on economic growth. In areas with high consumption intensity, it is not recommended to strongly promote renewable energy industry development. Thus, Hypothesis 1 is verified.

Second, energy transition also has a significant threshold effect, with a threshold value of 6.456. When ET ≤ 6.456, there is a significant negative impact; when ET > 6.456, the impact is significantly positive. This may be because, in the early stage of transition, the proportion of renewable energy consumption is small. Given this small scale, it cannot have a significant impact, mainly because the production and application of renewable energy involve low technologies and low utilization efficiency; in this case, the cost is higher than the economic benefit, thus posing a greater burden on the economy. When this proportion reaches a certain scale, the energy transition also enters a mature period, the renewable energy technology becomes relatively mature, the cost decreases, and the utilization efficiency greatly improves. Therefore, renewable energy can reduce environmental pollution without burdening the economy, which is conducive to economic growth. Thus, Hypothesis 2 is verified.

Third, emerging technologies also exert a significant threshold effect, with a threshold value of 1.367. When NT ≤ 1.367, there is a significant negative impact; when NT > 1.367, the impact is significantly positive. The reason may be that in the early stage of the development of emerging technologies, the cost of R&D is relatively high, R&D may fail, and it will take a long time for the emerging technologies to be applied after they are developed. This condition places a greater burden on the economy. Therefore, the development of technologies and economic growth have a negative correlation. With the maturity of technological R&D, the efficiency improvement brought about by emerging technologies can bring greater economic benefits. Emerging technologies help the renewable energy industry to reduce costs and improve the renewable energy efficiency. In this way, there will be no economic burden in the process of transformation, and these technologies will facilitate the relationship between energy transformation and economic growth. Thus, the development of emerging technologies at this stage can significantly improve the level of economic growth. Hence, Hypothesis 3 is verified.

## 5. Conclusions and Recommendations

### 5.1. Conclusions

This study investigates energy transition issues in the context of sustainable development, explores the panel threshold test method and the nonlinear impact of renewable energy consumption on economic growth, accurately identifies various factors that lead to the nonlinear impact, and verifies its threshold effect. The conclusions are as follows.

(1) Overall, the impact of renewable energy consumption on real GDP is negative, indicating that China’s current energy structure transformation strategy to increase renewable energy consumption incurs certain economic costs. However, in the long run, this negative impact will become positive as the level of technological innovation of renewable energy increases, the cost of renewable energy further decreases, and the increase in renewable energy consumption results in dynamic economies of scale and learning-by-doing effects.
(2) The regression results of the threshold model reveal a significant threshold effect on energy consumption intensity, and the threshold value is approximately 3.213. When EI ≤ 3.213, renewable energy consumption has a significantly positive impact on economic growth; when EI > 3.213, the impact is significantly negative. This shows that when the energy consumption intensity is low, economic growth can be promoted; however, when the energy consumption intensity is high, the economic cost of renewable energy consumption is also high.

Energy transition also has a significant threshold effect, with a threshold value of approximately 6.456. When ET ≤ 6.456, renewable energy consumption has a significantly negative impact on economic growth; when ET > 6.456, the impact is significantly positive. In the early stage of transformation, the cost of transformation is higher than the benefits, which will place a greater burden on the economy when economies of scale cannot be achieved. When the energy transformation reaches a certain scale, economies of scale will be achieved, and economic growth will be promoted.

There is also a significant threshold effect for emerging technologies, with a threshold value of approximately 1.367. When NT ≤ 1.367, renewable energy consumption has a significantly negative impact on economic growth; when NT > 1.367, the impact is significantly positive. The main reason for this is that the R&D of emerging technologies requires a large amount of capital investment in the early stage and thus causes a greater burden on economic growth because of the uncertainty of technology application. At this time, R&D investment is higher than the economic benefits. When technology R&D is relatively mature, the efficiency gains brought about by emerging technology outweigh the increase in cost, thus boosting economic growth.

### 5.2. Recommendations

Promote industrial technological progress. Technological progress can significantly improve energy efficiency. Therefore, it is necessary to support enterprises to improve their production technology through independent innovation or imitation innovation and to enhance the ability of technological innovation and the independent R&D of enterprises. Investments in social scientific research funds and subsidies for scientific and technological innovation should also be increased, along with the enthusiasm of enterprises for scientific and technological R&D. Moreover, the implementation of scientific research results should be improved, the upgrading of industrial technology should be accelerated, and scientific and technological innovation should be used to improve the energy efficiency. For regions with high energy endowments, the location advantage lowers their energy costs, resulting in insufficient government attention to energy efficiency improvement. Therefore, attention should be paid to energy efficiency assessment, the consideration of extractive industries, and the extension of related industrial chains, such as raw materials.

Coordinate regional economic growth and industrial energy consumption and promote the healthy development of the region. On the one hand, this approach advocates for the upgrading of industrial structures; accelerates high-tech industry development and service industries; increases the share of the tertiary industry; reduces industries with high energy consumption (e.g., steel and coal); and encourages the development of biomedicine, new energy vehicles, software and information services, and so on. On the other hand, it promotes a green and low-carbon lifestyle, increases investment in the construction of public transportation facilities, encourages residents to travel with consideration of green and low-carbon consumption, and jointly promotes regional economic growth.

Improve China’s energy consumption structure and increase renewable energy consumption. This study finds that in the long run, renewable energy consumption has a significant effect on China’s economic growth. Therefore, this study proposes to strengthen the use of renewable energy in China. China has huge potential for the development of renewable energy. Actively developing renewable energy will help to improve the energy supply and consumption structure and ensure that the energy efficiency increases, thereby guaranteeing the sustainable development of the economy. Following the principles of adapting measures to local conditions, rational layout, cost–benefit, and energy complementarity, the priority implementation of renewable energy industries includes hydropower, nuclear power, solar energy, wind energy, and geothermal energy.

Increase public awareness. The role of publicity should be emphasized—that is, the significance of renewable energy development, particularly for residents’ lives and consumption, should be publicized. In this regard, the Internet, television, radio, newspapers, and other media should be utilized to popularize the economic concept of green consumption, and advocate for diligence and frugality; publicize the significance of low-carbon consumption, energy conservation, and emission reduction; advocate for ecological civilization; and allow the public to realize that renewable energy development has an impact on their lives. Measures should encourage public welfare organizations related to the environment, life safety, and other factors to participate in the publicity of the significance of renewable energy development so that the public can understand it comprehensively.

Promote the reform of the energy system and mechanism to realize low-carbon energy transformation. The decisive role of the market in the allocation of energy resources should be brought into full play, the market-oriented reform of key areas and links should be deepened, and efforts should be made to solve the problem of an imperfect market system. Moreover, low-carbon energy transition should be promoted to provide guarantees. Reforms in the energy sector should be intensified, a competition mechanism for fair access should be introduced, and the diversification of investment in the energy sector should be promoted. Consequently, the interest relationship with consumers will fully reflect the commodity attributes of energy. Moreover, the construction of a national carbon emission trading market should be accelerated, market-oriented mechanisms should be used to reduce the production costs of energy companies, and a reasonable carbon price mechanism should be formulated. Other approaches may include the handling of green energy-saving dispatching and its relationship with the electricity market mechanism, the introduction of supporting policies to support green electricity, and the provision of institutional guarantees for low-carbon energy transformation.

## Figures and Tables

**Table 1:** Summary of literature.

Main Opinions	Authors
Increased consumption of renewable energy will boost economic growth	Charfeddine and Kahia (2019) [18]; Khan et al. (2020) [19]; Topcu and Tugcu (2020) [20]; Apergis and Salim (2015) [21]; Markandya et al. (2016) [22]; Zafar et al. (2020) [23]; Odhiambo (2009) [24]; Naseri et al. (2016) [25]; Zrelli (2017) [26]
Increased consumption of renewable energy at the expense of economic growth	Shahbaz et al. (2020) [27]; Maji et al. (2019) [28]; Han et al. (2020) [29]; Bhattacharya et al. (2016) [30]; Ocal and Aslan (2013) [31]; Qi and Li (2017) [32]
The increase in renewable energy consumption does not have a significant impact on economic growth	Payne (2009) [33]; Menegaki (2011) [34]; Bao and Xu (2019) [35]; Chang et al. (2015) [36]; Bulut and Muratoglu (2018) [37]; Yu and Hwang (1984) [38]; Kraft and Kraft (1978) [39]; Long (1997) [40]; Yu (1992) [41]
There are regional differences in the impact of renewable energy consumption on economic growth	Al-mulali et al. (2013) [42]; Yao and Zhang (2019) [43]; Yan et al. (2022) [44]; Qi and Li (2017) [32]; Guo and Cai (2022) [45]; Chen and Ye (2021) [46]

**Table 2:** Descriptive statistics of variables.

Variable	Observed Value	Mean	Standard Deviation	Minimum	Maximum
lnY	390	5.584	1.054	3.123	11.453
lnREC	390	5.563	1.432	0.775	8.994
lnNREC	390	7.642	0.963	3.222	8.052
lnEI	390	1.785	2.008	0.143	22.342
lnET	390	5.332	9.032	1.123	44.571
lnNT	390	0.456	0.421	0.216	1.563
lnK	390	6.432	1.776	3.664	9.042
lnL	390	5.996	0.984	5.649	11.531

**Table 3:** Single integer test.

Variable	LLC Test	IPS Test	Fisher-ADF Test	Fisher-PP Test
	t-Statistics	t-Statistics	t-Statistics	t-Statistics
lnY	−7.013 ***	−5.326 ***	131.095 ***	154.411 ***
lnREC	−13.124 ***	−10.987 ***	221.783 ***	277.932 ***
lnNREC	−8.179 ***	−3.876 ***	78.492 ***	144.342 ***
lnEI	−127.063 ***	−41.562 ***	212.875 ***	245.872 ***
lnET	−12.543 ***	−10.969***	166.324 ***	205.772 ***
lnNT	−10.223 ***	−8.128 ***	168.723 ***	211.657 ***
lnK	−9.223 ***	−16.165 ***	115.325 ***	217.043 ***
lnL	−16.127 ***	−18.033 ***	161.287 ***	228.619 ***

Note: *** represents a significance level of 1%.

**Table 4:** Cointegration test results.

Testing Method	Test Hypothesis	Statistics	Statistical Value
Kao Test	Ho: There is no cointegration relationship (ρ = 1)	ADF	−0.345 ***
Pedroni Test	Ho: ρi = 1 H1: (ρi = ρ) < 1	Panel v statistic	−1.347
		Panel rho statistic	2.568
		Panel PP statistic	−0.679 ***
		Panel ADF statistic	−1.022 ***
	Ho: ρi = 1 H1: ρi < 1	Group rho statistic	4.134
		Group PP statistic	−6.142 ***
		Group ADF statistic	−3.889 **

Note: ** and *** indicate significance at the 5% and 1% levels, respectively.

**Table 5:** Threshold effect test results.

Threshold Variable	Single Threshold Model		Double Threshold Model		Threshold Estimate	95% Confidence Interval
	F Value	*p* Value	F Value	*p* Value		
EI	1.281 ***	0.002	3.93	0.34	3.213	[2.981, 3,442]
ET	5.433 ***	0.001	9.442	0.23	6.456	[3.232, 24.283]
NT	34.722 ***	0.002	46.843	0.13	1.367	[1.142, 1.789]

Note: *** represents significant regression results at 1%.

**Table 6:** Regression estimation results.

Variable	Threshold Model			System GMM
	EI	ET	NT	
$\ln REC_{it}\cdot I\left\{q_{it}\leq \gamma \right\}$	0.0234	−0.0683 ***	−0.0187 ***	
$\ln REC_{it}\cdot I\left\{q_{it}>\gamma \right\}$	−0.1023	0.026 ***	0.0341 ***	
lnREC				−0.0485 ***
$lnY_{i,t-1}$	0.1322 ***	0.1588 ***	0.2384 ***	0.0446 ***
lnK	0.5432 ***	0.5192 ***	0.6534 ***	0.1293 ***
lnL	0.4387 **	0.4493 ***	0.5128 ***	0.3293 ***
lnNREC	0.0763 ***	0.0462 *	0.0873 **	0.0388 **
Constant	−4.002 ***	−4.4422 ***	−4.7832 ***	−3.2032 ***
Observations	390	390	390	390

Note: ***, **, * indicate significance at the 1%, 5% and 10% confidence levels, respectively.

## Data Availability

The data used to support the findings of this study are available from the corresponding author upon request.
